# Latest Advances on the Synthesis of Linear ABC-Type Triblock Terpolymers and Star-Shaped Polymers by RAFT Polymerization

**DOI:** 10.3390/polym13111698

**Published:** 2021-05-22

**Authors:** Athanasios Skandalis, Theodore Sentoukas, Despoina Giaouzi, Martha Kafetzi, Stergios Pispas

**Affiliations:** 1 Department of Materials, Imperial College London, London SW7 2AZ, UK; a.skandalis@imperial.ac.uk; 2 Center of Polymer and Carbon Materials, Polish Academy of Sciences, PL-41-819 Zabrze, Poland; tsentoukas@cmpw-pan.edu.pl; 3 Theoretical and Physical Chemistry Institute, National Hellenic Research Foundation, 11635 Athens, Greece; dgiaouzi@gmail.com (D.G.); mkafetzi91@gmail.com (M.K.)

**Keywords:** ABC triblock terpolymers, star polymers, RAFT polymerization, synthesis, self-assembly

## Abstract

This review article aims to cover the most recent advances regarding the synthesis of linear ABC-type triblock terpolymers and star-shaped polymers by RAFT polymerization, as well as their self-assembly properties in aqueous solutions. RAFT polymerization has received extensive attention, as it is a versatile technique, compatible with a great variety of functional monomers and reaction conditions, while providing exceptional and precise control over the final structure, with well-defined side-groups and post-polymerization engineering potential. Linear triblock terpolymers synthesis can lead to very interesting novel ideas, since there are countless combinations of stimuli/non-stimuli and hydrophilic/hydrophobic monomers that someone can use. One of their most interesting features is their ubiquitous ability to self-assemble in different nanostructures depending on their degree of polymerization (DP), block composition, solubilization protocol, internal and external stimuli. On the other hand, star-shaped polymers exhibit a more stable nanostructure, with a distinct crosslinked core and arm blocks that can also incorporate stimuli-responsive blocks for “smart” applications.

## 1. Introduction

Over the past 20 years, reversible addition-fragmentation chain transfer (RAFT) polymerization [1,2,3,4,5], a controlled/living radical polymerization method, has made a huge impact and has become an invaluable tool in the hands of synthetic polymer chemists by making possible the synthesis of previously unattainable polymers with complex macromolecular architectures such as block copolymers [6], star polymers [7] and hyperbranched polymers [8,9]. When compared to other controlled polymerization techniques, the RAFT process offers certain advantages, such as the synthesis of well-defined polymers with well-controlled molecular weights and molecular weight distributions in combination with the high compatibility with a wide variety of functional monomers (styrene, butadiene, (meth)acrylates, (meth)acrylamides, etc.) [10,11]. Nonetheless, RAFT is robust under large number of experimental conditions, even in aqueous media [12,13]. Other polymerization methods like atom transfer radical polymerization (ATRP) [14,15], free radical emulsion polymerization [16,17] and multi-component polymerization (MCP) [18,19] can also be performed in aqueous systems, provided that the suitable ligand is selected. Additionally, a purification process is needed for ATRP catalyst removal.

The controlled character of the RAFT process is derived from the presence of a chain transfer agent (CTA, RAFT agent) and the successful formation of polymers with low molecular weight distributions depends on the choice of the suitable CTA [3]. Typical RAFT agents include dithioesters, xanthates, trithiocarbonates and dithiocarbamates [3,20,21].

**Scheme 1:**
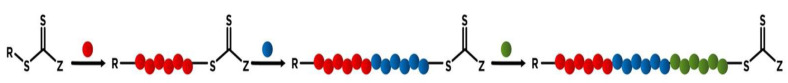
Schematic representation of the synthesis of ABC-type triblock terpolymers via sequential RAFT polymerization. The red, blue and green dots stand for the three different monomers.

Linear triblock copolymers of the ABC-type are a particularly interesting class of polymers due to the incorporation of three different blocks with different properties in their macromolecular chain [22]. Nevertheless, the design and synthesis of such polymers is always challenging. The versatility and the controlled/living character of RAFT polymerization, as well as the compatibility with a plethora of functional monomers, renders RAFT as one of the most promising polymerization methods for the synthesis of ABC triblocks [23]. In comparison with conventional AB 2eblock copolymers or ABA copolymers, the unique chemical structure of ABC triblock terpolymers adds more flexibility in terms of properties and gives the opportunity for more tailored final polymers. There is the potentiality of incorporating more than one block bearing different functional groups or blocks that respond to internal or external stimuli at the same time. It is this feature of ABC terpolymers that leads to the formation of exciting self-assembled nanostructures in aqueous solutions [24,25] such as core–shell corona structures [26], micelles with segregated coronas [27,28], multicompartment micelles [29], Janus particles [30] and several other morphologies. ABC triblocks by RAFT are normally synthesized by sequential addition of monomers (Scheme 1), with purifications taking place after the completion of each step [31].

Star polymers are another highly notable category of polymers with unique macromolecular architectures [32]. They are highly functional materials that consist of linear arms linked together to one central core and present scientific interest because of their remarkable solution properties with lower viscosity compared to their linear counterparts as well as their unique topological structures [33]. Till recently, the main approach for the synthesis of star polymers used to be anionic or cationic polymerization techniques [34]. However, the experimental conditions followed are challenging and eventually the star polymers synthesized via these synthetic strategies display severe limitations as far as the number of compatible monomers which can be incorporated into the macromolecule and by extension their usage in several applications may be compromised due to reasons involving potential scale up of the synthesis [35]. The progress of living radical polymerization methodologies such as RAFT and ATRP has provided an outstanding alternative for the synthesis of well-defined star-shaped polymers and rendered the preparation of such polymers quite feasible [36,37,38,39,40]. When RAFT polymerization is utilized for the synthesis of star polymers, there are two main strategies that can be followed, the “arm first” approach and the “core first” approach, as is the case with other controlled polymerizations. The “arm first” method involves the attachment of several linear polymer chains (arms) to a central point (core). Multifunctional low molecular weight coupling agents or crosslinkers are typically used as the star core. Oppositely, the “core first” method requires the synthesis of a multifunctional crosslinked core, in the active initiating sites of which, the development of linear polymers chains takes place. Star polymers are particularly attractive materials in the field of biomedical applications and more specifically they can be of use for tissue engineering and antibacterial biomaterials, as well as for diagnosis and drug/gene delivery applications [35,41].

The scope of this review is to highlight the most recent innovations and achievements regarding the synthesis of linear ABC triblock terpolymers and star-shaped polymers, employing RAFT polymerization methodologies. Moreover, we will briefly refer to the self-assembly properties of these polymers mainly in aqueous solutions as a first step towards applications.

## 2. ABC-Type Linear Triblock Terpolymers

### 2.1. General Synthetic Strategies for ABC Triblock Copolymers by RAFT

Up to this point, RAFT polymerization has generated precise synthesis of ABC-type triblock terpolymers, incorporating a wide variety of monomers bearing side groups of choice, as well as controlled molecular weights and narrow molecular weight-distribution. Factors like the block sequence and the composition of the final macromolecules can influence the nanostructures formed in solutions.

Indeed, Altintas et al. [42] reported the synthesis of ABC triblock terpolymers bearing primary alkyl bromide on block A, protected alkyne on block B and protected hydroxyl pendant groups on block C. The authors proceeded in post polymerization functionalization of the terpolymer with Hamilton wedge (HW) for block A, benzene-1,3,5-tricarboxamide (BTA) for block B and cyanuric acid (CA) for block C. It should be mentioned that the dithioester groups of the RAFT agent were removed by radical-induced reduction after the fabrication of the initial triblock.

In the same manner, Germack and Wooley [43] have presented the synthesis of ABC and ACB triblock terpolymers composed of tert-butyl acrylate (tBA), isoprene (Ip) and styrene (St) blocks, which are susceptible to post-polymerization functionalization. The synthesis started by creating the macro-chain transfer agent (mCTA) by polymerizing tBA utilizing a synthesized S-1-dodecyl-S′-(r,r′-dimethyl-r′-acetic acid)trithiocarbonate CTA agent, which was used as the initial block for the synthesis of P(tBA)_53_-*b*-P(Ip)_48_-*b*-P(St)_71_ and P(tBA)_53_-*b*-P(St)_46_*-b*-P(Ip)_133_ terpolymers with controlled molecular weights. Both triblock terpolymers presented rather narrow molecular weight distributions (1.3 and 1.5), depicting the ease-of-use and good control of the RAFT technique over the polymerization of all three monomers. 

Mixtures of solvents can be also utilized in RAFT, as Ghamkhari et al. [44] have shown with the synthesis of poly (2-hydroxyethyl methacrylate)-b-poly(*N*-isopropyl acrylamide)-b-poly[(*N*-4-vinylbenzyl), *N*,*N*-diethylaminehydrochloride] (PHEMA-b-PNIPAM-b-PVEAH) in a water/methanol mixture, with the use of 4-cyano-4-[(phenylcarbothioyl) sulfanyl] pentanoic acid as CTA. The triblock terpolymers exhibit strong anti-yeast and anti-microbial properties against *S. aureus*, *B. cereus*, *C. albicans* and *E. coli*.

The degree of polymerization of each block in ABC terpolymers seems to play an important role in the final nanostructures according to Xu et al. [45]. The group published the preparation of epoxy-functionalized poly (glycerol monomethacrylate)-*b*-poly (2-hydroxypropyl methacrylate)-*b*-poly(glycidylmethacrylate), (PGMA-*b*-PHPMA-*b*-PGlyMA) triblock terpolymer vesicles, synt hesized by RAFT polymerization. More specifically, PGMA was polymerized in ethanol with 4-cyano-4-(ethylthiocarbonothioylthio) pentanoic acid as the CTA and the homopolymer was utilized as macro-CTA for the polymerization of PHEMA via enzyme-initiated aqueous RAFT dispersion polymerization in ambient conditions, resulting to vesicular structures. The PGMA-*b*-PHPMA vesicles were used as seeds for the polymerization of GlyMA via seeded RAFT emulsion polymerization at room temperature, as the GlyMA monomer is insoluble in water. It was demonstrated that as the molecular weight of the PGlyMA block increased, the vesicles were becoming rougher and that the PGMA-b-PHPMA-b-PGlyMA triblock terpolymer vesicles could be utilized as Pickering emulsifiers for hexane-in-water emulsions.

Having this fact in mind, Zhang et al. [46] have presented a very interesting idea to synthesize ABC triblock terpolymers structure with the use of acrylated nucleobase containing monomers via RAFT polymerization. In that work, the group prepared three poly(thymine acrylate)-*b*-poly(n-butyl acrylate)-*b*-poly(adenine acrylate) with different DPs, mediated by the 2-cyano-2-propyl dodecyl trithiocarbonate as CTA. The nucleobase terpolymers tend to form lamellar and cylindrical structures in the bulk phase, while present excellent thermal and mechanical properties, which originate from the strong hydrogen bonding between adenine and thymine molecules and could be regulated by simply modifying the DP of each block.

Differences in block position have been presented by Messerschmidt et al. [47] with an interesting approach of anchoring amphiphilic triblock copolymers onto textile surfaces enabling them to withstand dirt and present better cleaning properties. In this manner, the group proceeded with the synthesis of two triblock copolymers, consisting of a hydrophobic poly (tert-butylstyrene-co-n-hexyl acrylate) P(tBS-co-nHA) block, a poly(*N*-acryloxysuccinimide) (PNAS) block, used for anchoring, and a poly(*N*,*N*-dimethylacrylamide) (PDMA) hydrophilic block. The polymerization was mediated with the use of S-methoxycarbonylphenylmethyl dithiobenzoate (MCPBD) CTA due to its ease of synthesis and the controlled polymerization obtained for the particular monomers. The difference between the two terpolymers lies in the position of the anchor block, which is placed either in the middle, or at the end of the triblock terpolymer chains. The resulting terpolymer conformations on the surface of textiles lead to the extension of relevant hydrophilic or hydrophobic blocks, depending on the environmental conditions.

RAFT polymerization can be also used in combination with other techniques as shown by Chen et al. [48] who prepared poly(oligoamide 11)-*b*-poly(lauryl methacrylate)-*b*-poly(methyl methacrylate) (OPA11-*b*-PLMA-*b*-PMMA) nanostructures. The group initially created the macro-CTA by melt polycondensation of oligoamide 11 and functionalizing it with 4-cyano-4-(phenylcarbonothioylthio) pentanoic acid (CPADB), while PLMA and PMMA were prepared by RAFT polymerization. Structural studies have revealed a vesicle-nanoparticle network consisting of a PLMA core, OPA 11 and a PMMA corona.

Synthesis and self-assembly of the block copolymers at the same time can be also achieved in order to study different formations in function with the degree of polymerization, as shown by Marble et al. [49] The group prepared poly(glycerol monomethacrylate)-*b*-poly(2-hydroxypropyl methacrylate)-*b*-poly(benzyl methacrylate) (PGMA-*b*-PHPMA-*b*-PBzMA) triblock terpolymers via polymerization induced self-assembly (PISA) RAFT technique which was mediated by the use of 2-cyano 2-propyl benzodithioate (CPDB) as CTA. The resulting molecular weight dispersities were rather narrow for all the samples, depicting a controlled polymerization process. Lower DP of PBzMA block leads to large worm-like structures, while further polymerization up to a point creates uniform spheres. Additionally, the terpolymers present low-sheer emulsifying ability when droplets of n-dodecane are introduced into their aqueous solutions.

The polymerization of the third block in a non-selective solvent can lead to interesting self-assembled nanostructures, as presented by Samanta et al. [50] that have utilized a modified surfactant-free RAFT-mediated miniemulsion polymerization to synthesize poly[2-(methacryloyloxy)ethyl ammonium chloride]-*b*-poly(n-butyl acrylate)-*b*-poly(isobornyl acrylate) (PMTAC-*b*-PBA-*b*-PIBA) ABC triblock terpolymers. The PMTAC macro-CTA was synthesized in water containing silica nanoparticles, utilizing 2-(dodecylthiocarbonothioylthio)-2-methylpropionic acid (DTMPA), while the third block was polymerized in a miniemulsion, forming raspberry-like particles. These nanostructures proved to have anti-icing and antibacterial properties and could be applied onto cotton fabric, paper or even glass, enabling them to be functional materials for polymer coatings or multifunctional paints.

### 2.2. Stimuli-Responsive ABC Triblock Copolymers

The macromolecular architecture of ABC-type triblock terpolymers offers the possibility for incorporation of one or more than one stimuli-responsive block in the same polymer. Thermo and pH-responsive monomers have been utilized in the synthesis of a considerable number of triblock terpolymers using RAFT polymerization, since they present interesting functionalities in terms of self-assembly, that in turn make them interesting as potential drug/protein nanocarriers for targeted delivery with controlled-release ability.

Pal et al. [51] published the synthesis of the pH- and thermo- responsive poly (*N*-isopropylacrylamide-*b*-n-butyl acrylate-*b*-4-vinylpyridine), (PNIPAM-*b*-PBA-*b*-P4VP) triblock terpolymers in DMF, with cyanomethyl dodecyl trithiocarbonate as the CTA and azobisisobutyronitrile as the thermal initiator. The self-assembly properties of the terpolymers in aqueous solutions at pH = 3 and pH = 8 were also investigated. It was proven that the terpolymers form core–shell-corona micelles with P4VP cores, PBA shells and PNIPAM coronas at temperatures below the LCST of PNIPAM and at pH > 4.7. When dissolved in chloroform, the PNIPAM-*b*-PBA-*b*-P4VP organize into globular morphologies with PNIPAM, PBA and P4VP as the inner, intermediate and outer layers, respectively.

**Scheme 2:**
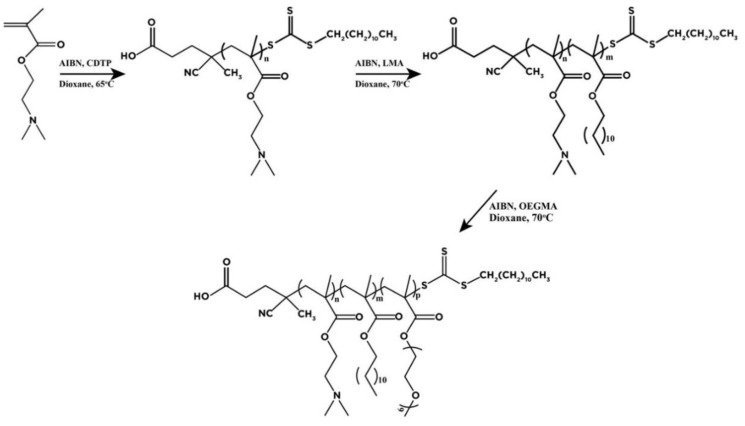
Synthesis of PDMAEMA-b-PLMA-b-POEGMA triblock copolymers by sequential RAFT polymerization [52].

The synthesis of a series of poly[2-(dimethylamino)ethyl methacrylate-b-lauryl methacrylate-b-(oligo ethylene glycol)methacrylate], (PDMAEMA-b-PLMA-b-POEGMA) triblock terpolymers via sequential RAFT polymerization was presented by Skandalis and Pispas [52], utilizing 4-cyano-4-[(dodecylsulfanylthiocarbonyl)sulfanyl] pentanoic acid as the CTA, starting from the preparation of PDMAEMA homopolymer which was later on used as the macro-CTA for the synthesis of the PDMAEMA-b-PLMA diblock copolymer. The diblock was used as the macro-CTA for the chain extension with OEGMA, resulting to the final triblock terpolymers (Scheme 2). A typical quaternization reaction with methyl iodide followed, in order to convert the tertiary amine groups of PDMAEMA into quaternary amine groups with permanent positive charges giving QPDMAEMA-b-PLMA-b-POEGMA cationic terpolymers. Both the amine and quaternized terpolymers self-assemble into spherical micelles with PLMA cores and (Q)PDMAEMA/POEGMA mixed coronas in water. The initial terpolymers do not present response to pH variations due to crowding effects in the micelle corona that originate from the presence of POEGMA blocks. The QPDMAEMA-b-PLMA-b-POEGMA micelles were utilized as non-viral vectors for complexation with DNA [53] and insulin [54], via electrostatic interactions, and the physicochemical properties of the complexes were studied in considerable detail as a function of components mixing ratio besides ionic strength of the aqueous solutions.

The same group reported on the synthesis of poly (n-butyl acrylate-*b*-N isopropylacrylamide-*b*-2-(dimethylamino) ethyl acrylate), (PnBA-*b*-PNIPAM-*b*-PDMAEA) double-responsive (temperature and pH) triblock terpolymers [55]. The PDMAEA amine groups were quaternized with CH_3_I to give triblock terpolymers with permanent positive charges. The effect of pH and temperature variations on the self-assembly properties of both the amine and quaternized terpolymers in aqueous solutions were investigated. It was observed that the PnBA-*b*-PNIPAM-*b*-PDMAEA terpolymers form spherical micelles with a tendency for intermicellar aggregation, even at room temperature conditions, while the spherical micelles of the quaternized terpolymers show lower or no tendency for aggregation (depending on the PDMAEA ratio), due to their higher solubility in water.

Temperature and pH- responsive triblock terpolymers composed of poly(ethylene oxide)-b-poly(*N*-isopropylacrylamide)-b-poly(acrylic acid), (PnBA-b-PNIPAM-b-PAA) by sequential RAFT polymerization in dioxane were presented by Papagiannopoulos et al. [56], using 2-dodecylsulfanylthiocarbonylsulfanyl-2-methylpropionic acid as the CTA. The self-assembly behavior as well as the response to temperature alterations of the terpolymers in aqueous solutions were investigated using scattering methods. Moreover, the group studied the effect of the addition of lysozyme, a positively charged protein, in the aqueous polymer solutions Electrostatic interactions with the oppositely charged PAA block at pH 7 were observed. The results demonstrate the formation of aggregates at room temperature, that transform into dense objects above the LCST of PNIPAM. The addition of lysozyme amplified the inter-aggregate interactions and led to significant increase in the size and scattering intensity, which becomes even more pronounced as solution temperature increases.

Giaouzi et al. [57] worked on the synthesis and self-assembly in H_2_O of poly(2-(dimethylamino)ethyl acrylate)-*b*-poly(*N*-isopropylacrylamide)-*b*-poly(oligo ethylene glycol methyl ether acrylate), (PDMAEA-*b*-PNIPAM-*b*-POEGA) triple hydrophilic and double-responsive (temperature and pH) triblock terpolymers. The synthesis was achieved by three-step RAFT polymerization in dioxane, involving 2-(dodecylthiocarbonothioylthio)-2-methylpropionionic acid as the CTA. The group also performed post-polymerization functionalization on the tertiary amine pendant groups of the PDMAEA polyelectrolyte block with iodomethane and 1-iodohexane (via quaternization reactions), as well as with 1,3-propanesultone (via sulfobetainization reaction). The self-assembly studies on the parent terpolymers revealed the formation of aggregates, which become more compact above the phase-transition temperature of PNIPAM, due to thermally induced aggregation and changes in the solvation state of the PNIPAM block. The quaternized triblocks form larger aggregates than the initial triblock through the whole temperature range and it was observed that the phase-transition temperature varies depending on the length of the hydrocarbon chain of the quaternizing agent. On the other hand, the zwitterionic terpolymers exhibit the opposite behavior and their aggregates show an increase in size as temperature increases.

The synthesis of thermo- and pH-responsive triblocks composed of poly(ethylene glycol)-*b*-poly(2-(2-methoxyethoxy) ethyl methacrylate-co-*N*-hydroxymethyl acrylamide)-*b*-poly(2-(diethylamino) ethyl methacrylate), (mPEG-*b*-P(MEO_2_MA-*co*-HMAM)-b-PDEAEMA) was investigated by Liu et al. [58]. In order to achieve that, they synthesized a PEG containing macro-RAFT agent which was utilized for the synthesis of the P(MEO_2_MA-*co*-HMAM) random middle block. Following this, the mPEG-*b*-P(MEO_2_MA-*co*-HMAM) diblock was used as macro-CTA for the chain extension of DMAEMA in dioxane, resulting to the final ABC triblocks. The terpolymers form core–shell micelles when inserted in aqueous solutions of neutral pH, with mixed P(MEO_2_MA-*co*-HMAM) and PEG shells, and PDEAEMA cores. In acidic pH, the terpolymers form micelles with P(MEO_2_MA-*co*-HMAM) cores and mixed PDEAEMA/PEG coronas. 

Mahmoodzadeh et al. [59] prepared poly(acrylic acid)-*b*-poly(*N*-isopropylacrylamide)-*b*-poly(ε-caprolactone)-SH(PAA-*b*-PNIPAM-*b*-PCL-SH) ABC triblock copolymers by combining ring-opening (ROP) and RAFT polymerization. The group started with the synthesis of bis(2-hydroxyethyl) disulfide, which was used as an initiator for the ring opening of ε-caprolactone in order to prepare the (PCL-S)_2_ precursor block. For the next step, 4-cyano-4-[(phenylcarbothioyl)sulfanyl]pentanoic acid was utilized for the preparation of the (PCL-S)_2_ macro-CTA, which was used for the sequential polymerization of the remaining PNIPAM and PAA blocks. The final step comprises of the side-group modification to produce thiol-end capped PAA-*b*-PNIPAM-*b*-PCL-SH triblock terpolymers. Self-assembly studies in aqueous solutions show a temperature and pH responsive behavior that results in nanostructures of different size.

Guragain et al. [60] studied the effect of solution pH on the polymerization of poly (ethylene oxide-b-(2-dimethylamino) ethyl methacrylate-b-(2-hydroxypropyl methacrylate)) via visible light-mediated RAFT polymerization. For that purpose, 2-dodecylthiocarbonothioylthio-2-methylpropionate was used as the CTA and tris(2,2′-bipyridyl) dichlororuthenium(II) hexa- hydrate Ru(bpy) (3Cl_2_·6H_2_O) as the radical initiator. Self-assembly studies have shown that different morphologies and structures can be obtained by regulating the solution pH and the degree of polymerization (DP) of the PHPMA hydrophobic block.

The self-assembly properties of double thermo-responsive triblock terpolymers were studied by Ye et al. [61] who presented the synthesis of poly(di(ethylene glycol)ethyl ether acrylate)-b-poly(*N*,*N*-dimethylacrylamide)-b-poly(*N*-vinylcaprolactam) (PDEGA-b-PDMA-b-PVCL). The terpolymers were synthesized by a typical RAFT polymerization procedure with 2-ethylsulfanylthiocarbonylsulfanylpropionic acid methyl ester as the CTA in a water-ethanol solution mixture. The thermal phase transitions of the terpolymers were studied and it was revealed that they exhibit two LCST transitions due to the existence of both PDEGA and PVCL blocks in their macromolecular chain. Micelles with PDEGA cores and PVCL/PDMA outer layers are formed when the LCST of PDEGA is reached. Above the LCST of PVCL aggregates of micelles are formed that shrink in size as temperature increases further and PVCL dehydrates.

In the same context, Mäkinen et al. [62] utilized a PEO containing macro-RAFT agent in order to synthesize temperature-responsive and triple hydrophilic ABC triblock terpolymers with different sequence and block lengths. The other two blocks incorporated were *N*-acryloylglycinamide (NAGA) and *N*-isopropylacrylamide (NIPAM), resulting in PEO-b-PNAGA-b-PNIPAM and PEO-b-PNIPAM-b-PNAGA triblocks. Investigations of the aqueous solutions of the terpolymers showed that they exhibit both UCST and LCST transitions, promoting the formation of aggregates below the UCST and above the LCST. The aggregates are able to inverse their morphology upon heating and cooling. The chain length, as well as the block sequence have an effect on the thermo-responsive behavior of the terpolymer solutions.

Davaran et al. [63] reported the synthesis of poly [(2-succinyloxyethyl methacrylate)-b-(*N*-isopropylacrylamide)-b-[(*N*-4-vinylbenzyl),*N*,*N*-diethylamine]], [P(SEMA-b-NIPAM-b-VEA)] double-responsive triblock terpolymers. RAFT polymerization was employed for the synthesis of P(HEMA-b-NIPAM-b-VEA) precursor terpolymers and the hydroxy side groups of PHEMA block were then modified to succinyloxy groups through esterification with succinic anhydride. The terpolymers exhibit “schizophrenic” self-assembly behavior in aqueous solutions. In acidic pH, micelles are formed with PSEMA, PNIPAM and PVEA as cores, shells and coronas, respectively. Decrease in the size of the micelles was observed as the pH value was increased (ionization of PSEMA and deprotonation of PVEA) and at pH 10 the formed micelles are composed of PSEMA coronas, PNIPAM shells and PVEA cores. On the other hand, the size of the micelles decreased as temperature increased. The terpolymers were also examined as potential nanocarriers for the anti-cancer drug doxorubicin and showed sufficient drug loading capacity and satisfactory drug release.

Huang et al. [64] reported on the synthesis of multi-responsive poly(methyl methacrylate)-b-poly[*N*,*N*-(dimethylamino) ethyl methacrylate]-b-poly(*N*-isopropylacrylamide), (PMMA-b-PDMAEMA-b-PNIPAM), triblock terpolymers via consecutive RAFT polymerization in toluene, with 4-cyanopentanoic acid dithiobenzoate as the RAFT-agent and 2,2′-azobis (isobutyronitrile) as the radical initiator. The PDMAEMA block is both thermo and pH-responsive and the PNIPAM block is thermo-responsive. The terpolymers form core–shell micelles with PMMA cores and mixed PDMAEMA/PNIPAM coronas. The ability of the terpolymers to form gels at high temperatures and acidic pH was proven in this study.

Banerjee and Dhara [65] reported on the synthesis of two different temperature-responsive ABC-type triblock architectures by sequential RAFT in dioxane, using an in house synthesized PEG-based CTA. The resulting triblock terpolymers were of the types poly(ethylene glycol)-b-poly(*N*-isopropylacrylamide)-b-poly(t-butyl acrylate), (PEG-b-PNIPAM-b-PtBA), and poly(ethylene glycol)-b-poly(*N*-isopropylacrylamide)-b-poly-(glycidyl methacrylate), (PEG-b-PNIPAM-b-PGMA). Both types of terpolymers self-organized into aggregates when inserted in aqueous solutions. However, above the LCST of PNIPAM, PEG-b-PNIPAM-b-PtBA terpolymers form micellar structures, while PEG-b-PNIPAM-b-PGMA terpolymers undergo macroscopic phase separation under similar conditions. The group also investigated the effect of conversion of PtBA into PAA via hydrolysis and of PGMA epoxy-groups into diol via ring opening of epoxide. The PEG-b-PNIPAM-b-PAA and PEG-b-PNIPAM-b-PGMA-diol terpolymers both form vesicular structures above the LCST of PNIPAM.

Koonar et al. [66] employed a combination of anionic and RAFT polymerization methodologies for the synthesis of two poly(ethylene-alt-propylene)-b-poly(ethylene oxide)-b-poly(*N*-isopropylacrylamide-co-acrylic acid), (PEP-b-PEO-b-P(NIPAM-co-AA)), triblock terpolymers. Initially, PEP-b-PEO-CTA was synthesized by anionic polymerization and end functionalized to give PO-CTA, which was then used for the synthesis of the P(NIPAM-co-AA) block by RAFT polymerization. It must be noted that after obtaining the final triblock terpolymers, the authors removed the end groups coming from the CTA in order not to affect the solution properties of the terpolymers. Investigation of PEP-b-PEO-b-P(NIPAM-co-AA) aqueous solutions revealed the formation of micelles with PEP cores and PEO-P(NIPAM-co-AA) coronas at room temperature. The polymer solutions undergo aggregation and gelation upon heating, due to intermicellar association of the P(NIPAM-co-AA) block above the LCST.

Guang et al. [67] proceeded with the design and synthesis of a doubly thermoresponsive ABC triblock terpolymer system composed of poly(ethyleneglycol)-*b*-poly(2-(2-methoxyethoxy)ethyl methacrylate)-*b*-poly(2-(2-methoxy ethoxy) ethyl methacrylate-*co*-oligo(ethylene glycol) methyl ether methacrylate) (PEG-*b*-PMEO_2_MA-*b*-P(MEO_2_MA-*co*-OEGMA)). The group initially prepared the CTA S-1-dodecyl-S-(a,a-dimethyl-a′-acetic acid) trithiocarbonate in order to mediate the polymerization of every block, and molecular weight polydispersities in each step were rather narrow, ranging from 1.2 to 1.5. The synthesized triblock terpolymers self-assemble into micelles in aqueous solutions at 29 °C, while a temperature rise above this value was found to lead to gelation after a reversible two-step LCST transition.

In the same context, Li et al. [68] have achieved the synthesis of doubly thermoresponsive PNIPAM-*b*-PDMAEMA-*b*-PS and PDMAEMA-*b*-PNIPAM-*b*-PS triblock terpolymers in a methanol/water 4:1 ratio. The group also prepared the 4-cyano-4-(dodecylsulfanylthiocarbonyl)sulfanyl pentanoic acid (CDTPA) CTA agent from scratch, which was able to maintain a controlled polymerization for the synthesis of each block. The idea was to take advantage of the two LCST’s that these triblock terpolymers presented in order to manipulate the nanoparticle structure by disposing them to water and regulating the temperature in three different stages. Thus, dissolution in water leads to the formation of micelles with a PS core and PDMAEMA-PNIPAM corona. Temperature increase above the LCST of PNIPAM forces its chains to shrink near the core, leaving PDMAEMA chains extended. Further temperature increase just above the LCST of PDMAEMA invokes chain collapsing, while heating the solution even further leads to micelle aggregation and finally precipitation.

The synthesis of polystyrene-b-poly(*N*,*N*-dimethacrylamide)-b-poly(4-vinylpyridine) (PS-b-PDMA-b-P4VP) by the solvent-exchange protocol using seeded RAFT polymerization in water/ethanol 1:1 mixture has been reported by Huo et al. [69]. The group has synthesized 4-cyano-4-(ethylsulfanylthiocarbonyl)sulfanyl pentanoic acid (ECT) CTA to provide a controlled polymerization process and take advantage of its trithiocarbonate (TTC) terminal group. The authors observed the formation of well-defined corona-shell nanoparticles in this solvent mixture, while transferring the samples to pure deionized water forces the hydrophobic P4VP block to retract in the core, leaving PDMA as the corona. They also successfully regulated the nanoparticle structure from corona-core micelles to multicompartment nanoparticles by just increasing the degree of polymerization of P4VP block.

Manipulation of the final self-organized structures of ABCs in an aqueous solution by initially modifying the degree of polymerization of the PNIPAM block and regulating the solution temperature above the PNIPAM LCST was reported by Qu et al. [70] They synthesized poly(styrene)-*b*-poly(*N*,*N*-dimethylacrylamide)-*b*-poly(*N*-isopropylacrylamide) (PS-*b*-PDMA-*b*-PNIPAM) ABC triblock terpolymers in a 1:1 mixture of acetone/n-hexane, with the use of 4-cyano-4-(ethylsulfanylthiocarbonyl)sulfanyl pentanoic acid (ECT) as CTA. In this manner, shorter PNIPAM chains produce nanorods, while longer chains lead to nanospheres. At temperatures lower than the LCST, the structure resembles a micelle with PS as the core and PDMA-PNIPAM extended chains as the corona. By increasing the solution temperature above PNIPAM LCST, its chains collapse and turn towards the PS core, leaving the PDMA chains on the outer rim as the new corona.

### 2.3. Triblock Terpolymers Self-Assembly Properties in Solvent Exchange Protocols

Since each block of an ABC triblock terpolymer may by soluble in a different solvent, the solvent-exchange protocol can be utilized in order to enable tailoring of the self-assembly properties and the nanostructure formation in aqueous solutions.

Li et al. [71] developed α-norbornenyl polystyrene-b-poly(methyl acrylate)-b-poly(tert-butyl acrylate), (NB-PS-b-PMA-b-PtBA), ABC-type triblocks via sequential RAFT polymerization, using a norbornene-functionalized CTA. The NB-PS-b-PMA-b-PtBA terpolymers were used as triblock macromonomer for the preparation of PNB-g-(PS-b-PMA-b-PtBA) polymer brushes by ring opening metathesis polymerization (ROMP). Finally, the PtBA block was converted to PAA. The self-assembly behavior of the NB-PS-b-PMA-b-PtBA linear terpolymers and the PNB-g-(PS-b-PMA-b-PtBA) brushes were compared. When transitioned from DMF to water, the ABC-type triblocks form globular structures, while the brushes exhibited cylindrical morphologies.

Muslim et al. [72] presented the synthesis of poly(t-butyl acrylate)-*b*-poly(styrene)-*b*-poly(2-vinyl pyridine) (PtBA-*b*-PS-*b*-P2VP) triblock terpolymers and the manipulation of their self-assembly properties in solutions. The polymerization was mediated by the S-ethyl-S-(a, a-dimethyl-a′-acetic acid)trithiocarbonate (EDMAT), which was prepared in house. Molecular weight polydispersity was kept low at every step, with a final value of 1.22, depicting the controlled nature of the followed RAFT polymerization scheme. Self-assembly studies were conducted by refluxing the THF solution of the triblock terpolymers into different solutions of methanol and distilled water of pH 3. Spherical nanoparticles were formed in the case of methanol, while “core-compartmentalized” micelles were produced in water, with the particle diameter size range was from 22 to 46 nm.

Fernandez-Alvarez et al. [73] presented the synthesis of (poly(acrylic acid)-b-poly(4-hydroxystyrene)-b-poly[1-(4-(1-methyl-1,2-dicarba-closo- dodecaborane-2-yl methyl)-phenyl)ethylene] (PAA-b-PHS-b-PSC) triblock terpolymers with carborane pendant groups. The polymerization was mediated by 2-(dodecylthiocarbonothioylthio)-2-methylpropionic acid CTA and 2,2′-azo-bis-isobutyronitrile (AIBN) as the initiator. Self-assembly studies have shown that a transfer from a water/THF mixture to water leads to a structural transition from spherical to cylindrical micelles, while also presenting pH and photophysical properties, responding to pH and fluoride anions.

## 3. Star-Shaped Polymers

The design and synthesis of well-defined polymers with complex macromolecular architectures has been subject of increasing attention over the past decades. Amongst them, star polymers are highly functional materials that consist of linear arms linked together to one central core and present considerable scientific interest because of their remarkable solution properties, showing a lower viscosity compared to their linear counterparts, as well as their unique topological structures. [33] In past decades, the main synthetic approach for star polymers used to be anionic or cationic polymerization techniques [34]. However, the experimental conditions followed are challenging and eventually the star polymers synthesized via these synthetic strategies display limitations in their usage in several applications. [35] In recent years, with the progress of controlled radical polymerization methodologies, such as RAFT and ATRP polymerization techniques, the synthesis of star polymers has been substantially more accessible. On account of their highly versatility and synthetic ease, these polymerization methodologies have provided an outstanding alternative avenue for the development of well-defined star polymers [36,37,38,39].

In the following sections, we aim to highlight the most recent achievements in the RAFT synthesis schemes for star polymers. Particularly, we discuss the preparation of star polymers by using RAFT polymerization methodologies, which has gained considerable attention owing to the fact that it demonstrates significant advantages, including the compatibility with a great variety of functional monomers, the synthesis of well-defined polymers with controlled molecular characteristics and in comparison, with ATRP, the absence of non-biocompatible catalyst during the synthetic procedures [1,2,5,35]. A challenge in the preparation of star polymers by RAFT polymerization is related to the required chain transfer agents (CTA) synthesis, as well as the efficacy of the chain transfer procedures, which affects the structural integrity of the resulting polymer, including important aspects, such as polymer dispersity, control of arm number and length, and end-chain fidelity. Some of the significant factors required to take under consideration are the steric hindrance and congestion at the reactive sites (namely the thiocarbonylthio group) which influences the way in which the addition and fragmentation processes can take place, together with the diverse kinds of impurities derived from star–star coupling or linear polymers that can be produced during the polymerization procedure based on the envisaged mechanism [7].

The synthesis of star polymers via RAFT polymerization as described in the literature can be performed utilizing either a core-first or an arm-first approach. In the beginning, the core-first approaches were favored for the synthesis of star polymers via RAFT polymerization but in recent years, the scientific interest concentrates on the arm-first methodologies. This shift in interest may be attributed to the growing attention of the end group chemistry of the RAFT synthesized polymers and the appearance of efficient click chemistries [31].

## 3.1. “Core-First” Approach

The preparation of star polymers via the “core-first” approach by RAFT polymerization requires a compound composed of multiple thiocarbonylthio groups with appropriate structure, known as multifunctional chain transfer agents (RAFT agents), whereby the linear polymer chains (polymeric arms) will effectively grow. Among the polymer synthesis techniques, RAFT polymerization presents a unique feature concerning the core-first approach that can be further divided into two subcategories based on the orientation of the thiocarbonylthio moiety in the RAFT agent: the Z-group approach and the R-group approach [74,75,76,77]. The multifunctional RAFT agents contain both a nonfragmenting (Z-) and a fragmenting (R-) group and the core-first synthetic strategy is defined by which of these groups is conjugated to the central core. In the case of Z-group synthetic strategy, the thiocarbonyltio groups remain close to the core and the arms incur fragmentation and addition as the polymerization progresses. Contrarily, in the case of R-group synthetic route, the central core is basically the leaving group and the polymeric chains are polymerized outward from the central core [35].

**Scheme 3:**
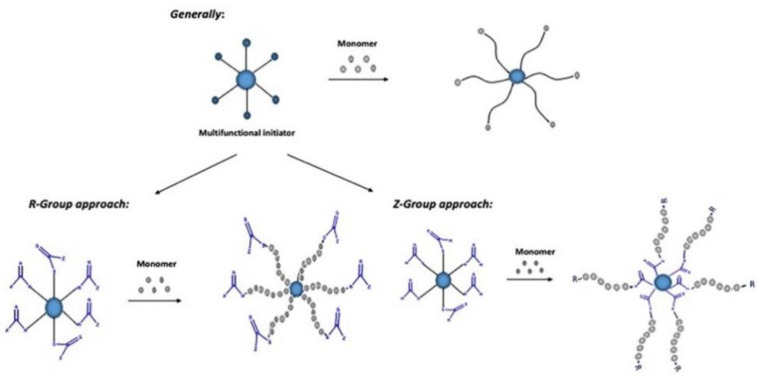
Schematic illustration of the “core- first” approach.

In the Z-group approach, the Z-groups are covalently linked to the core preventing the star–star coupling, a side reaction that may occur when utilizing the R-group approach. Star–star coupling is usually noticed in reversible-deactivation radical polymerization procedures when the core-first synthetic strategy is implemented, including the R-group approach of RAFT polymerization. This is taken place on account of the fact that the propagating radical moieties are placed at the end of each arm [78]. The R-group approach displays significant advantages, including the existence of the thiocarbonyltio moieties at the periphery of the stars, providing additional potentialities for postpolymerization functionalization reactions. Furthermore, if the side reactions regarding the star–star coupling could be circumvented this synthetic approach would provide the potential to synthesize high molecular weight star polymers having exact number of arms, which is too challenging to be achieved by implementing the Z-group approach [40,77]. A schematic illustration of core-first approach is presented in Scheme 3.

The most recent developments concerning the synthesis of star polymers via RAFT polymerization by utilizing the core-first approach are presented in the following. Cao et al. [79] followed the core-first approach in order to synthesize 2-, 3- and 4-arm temperature-responsive star polymers of poly (*N*-acryloylsarcosine methyl ether) [(PNASME)_n_] and poly(*N*-isopropylacrylamide) [(PNIPAM)_n_] by using a series of multifunctional CTAs (chain transfer agents). Linear counterparts of these polymers utilizing mono-functional CTA were synthesized for comparison purposes. Initially, they synthesized the chain transfer agents, specifically the mono-functional CTA butyltrithiomethyl benzene (1) and the multi-functional 1,2-bis-(butyltrithiomethyl)benzene (2), 1,3,5-tris-(butyltrithiomethyl)benzene (3) and (butyltrithiomethyl)benzene (4) CTAs. It must be noted that the similarity between the R-group and the Z-group in each CTAs renders them useful for the synthesis of star polymers with uniform arms. The linear and star polymers of (PNASME)_n_ and (PNIPAM)_n_ were synthesized with the similar procedures utilizing 2,2′-Azobis(2-methylpropionitrile) (AIBN) as radical initiator, the 1, 2, 3, 4 as chain transfer agents and dioxane as the polymerization solvent. All synthesized polymers (linear and stars) with different molecular weights displayed narrow molecular weight distributions that are in the range from 1.05 to 1.26 as determined by SEC. After the preparation of both linear and star polymers, they concluded that the polymerization of linear counterparts using mono-functional CTA proceeds faster than those using multi-functional CTA under similar reaction conditions. The possible reason was attributed to the less viscous medium in the polymerization solution of star polymers. Moreover, the authors conducted studies on the phase transition temperature (PTT) of the synthesized temperature-responsive polymers and they found that the PPT factor is correlated to the polymer topology. In particular, the star polymers of (PNASME)_n_ and (PNIPAM)_n_ have lower PPT and higher dehydration degree than the linear counterparts which increases as the number of arms increases. They deduced that the polymer topology is a significant parameter influencing the phase transition temperature of temperature-responsive polymers.

Another study regarding the synthesis of star polymers based on *N*-isopropylacylamide (NIPAM) via the “core-fist” approach was conducted by Bian et al. [80] They synthesized star diblock copolymers of poly(ε-caprolactone)-b-poly(*N*-isopropylacrylamide) (SPCLNIP) via RAFT polymerization using the R-group approach. Initially, they synthesized two-star polymers of poly(ε-caprolactone) (PCL) with four arms of different length having terminal hydroxyl groups (SPCL-OH) via controlled bulk ROP, utilizing pentaerythritol (PERT) as the multifunctional initiator at different molar ratios of [CL]/[PERT] and stannous 2-ethyl-hexanoate [(Sn (Oct)_2_] as the catalyst. Thereafter, the authors prepared the star macro-RAFT agent through esterification reaction of SPCL-OH and S-1-dodecyl-S0-(α, α′-dimethyl-α′′-acetic acid) trithiocarbonate (DDAT) RAFT chain transfer agent in the presence of N,N′-dicyclohexylcarbodiimide (DCC)/4-(dimethylamino)pyridine (DMAP). The end-functionalized star diblock copolymers (SPCLNIP) were produced using two different in molecular weight trithio-carbonate-terminated star poly(ε-caprolactone) (SPCL-DDMAT) as tetra functional macro-RAFT agents, NIPAM monomer and dioxane as the solvent. The polymerization was carried out at two different temperatures (85 °C and 100 °C) in order to examine the R-RAFT polymerization of NIPAM by utilizing the (SPCL-DDMAT) as star macro-RAFT agent. The extracted results demonstrated that the R-RAFT polymerization of NIPAM is a controlled polymerization method over a wide range of temperatures, despite the fact that it displayed an induction period at lower polymerization temperatures. No additional radical initiator was utilized to eliminate the radical termination reactions and all radicals are supposed to be generated by the thermal initiation of NIPAM. Two different in molecular weight star macro-RAFT agents SPCL_25_-DDMAT and SPCL_10_-DDMAT were used in order to scrutinize the polymerization of NIPAM under the same polymerization conditions. The results showed that the polymerization rate of NIPAM at 100 °C utilizing SPCL_10_-DDMAT star macro-RAFT agent was lower and the polymerization procedure was better controlled than those utilizing SPCL_25_-DDMAT star macro-RAFT agent. Bian et al. concluded that the difference on the polymerization rate was attributed to the variation of viscosity in their system as SPCL_25_-DDMAT with high molecular weight occupied higher viscosity than the SPCL_10_-DDMAT with low molecular weight under the same reaction conditions. SPCL_10_-DDMAT with high viscosity demonstrated a lower diffusion rate and as a result poor capability of transferring the propagating chain radicals in order to generate the dormant species. The molecular characteristics of the synthesized star polymers were determined by ^1^H NMR, ^13^C NMR and SEC. From SEC curves, tiny low-molecular weight tails were noticed with increasing conversion owing to the formation of linear PNIPAM chains when utilizing SPCL_25_-DDMAT as star macro-RAFT agent. On the contrary, linear PNIPAM chains were not detected when utilizing SPCL_10_-DDMAT. In addition, the thermal properties of the star polymers were examined by DSC and TGA, demonstrating that the amorphous PNIPAM chains destroyed the crystallinity of PCL segments and the Tg of PNIPAM segments increased as the PNIPAM chain lengths increased. Consequently, the temperature of polymerization as well as the molecular weights of star macro-RAFT agents significantly influenced R-RAFT polymerization and the side reactions regarding the star–star coupling can be avoided by appropriate selection of macro-RAFT agent. Further functionalization of trithiocarbonate groups placed at the periphery of star block copolymers is anticipated to render them useful as potential materials for biomedical applications.

Herfurth et al. [81] achieved the one-pot synthesis of amphiphilic star polymers via RAFT polymerization by using multifunctional chain transfer agents. For the synthesis of star polymers, they followed the R-group strategy in the core-first approach. They synthesized 2- arm linear polymers serving as reference and 3- and 4- arm star polymers made of *N*, *N*-dimethylacrylamide (DMA) monomer. At first, they produced the bifunctional CTAs: 1,3,5-tris(butylsulfanylthiocarbonylsulfanylmethyl)benzene (3C4-CTA), 1,2,4,5-tetrakis(butylsulfanylthiocarbonylsulfanylmethyl)benzene (4C4-CTA), 1,3,5-tris(dodecylsulfanylthiocarbonylsulfanylmethyl)benzene (3C12-CTA), 1,2,4,5-tetrakis(dodecylsulfanylthiocarbonylsulfanylmethyl) benzene (4C12-CTA), 1,3,5-tris(octadecylsulfanylthiocarbonylsulfanylmethyl)benzene (3C18-CTA), and 1,2,4,5-tetrakis(octadecylsulfanylthiocarbonylsulfanylmethyl)benzene (4C18-CTA). For the synthesis of all CTAs, they followed a general protocol using carbon disulfide and multifunctional benzyl bromides as the building blocks. They synthesized n-alkyl chains with lengths of 4, 12, 18 atoms of carbon as hydrophobic end groups so as to examine the effect of the hydrophobicity of these end groups on the aggregation behavior of the stars. A general protocol followed for all RAFT polymerizations of star polymers utilizing DMA as monomer, the above-mentioned CTAs for each polymerization procedure, 1,1′-azobis(cyclohexanecarbonitrile) as initiator and benzene as the solvent. They were able to synthesize amphiphilic star polymers based on hydrophilic DMA monomer presenting high polymerization yields and good control in an extended range of molar masses. The obtained results from SEC and UV/Vis measurements showed that the synthesized star polymers demonstrated narrow molar mass distributions (PDI ≤ 1.2) and high-end group functionality (~90%), respectively. The highly hydrophilic star polymers were capable of self-assembling in aqueous media due to the existence of hydrophobic end groups. Moreover, the combination of small angle neutron scattering, dynamic light scattering (DLS) and rheology experiments revealed that the length of the hydrophobic chain ends (stickiness), the length of hydrophilic block (maximum length for connection) and the arms number (functionality) affected the structure and the overall physical properties of the formed aggregates. Conclusively, they found that lower concentrations resulted in the formation of flower-like micelles while higher concentrations led to the formation of a transient micellar network. Additionally, the amphiphilic star polymers showed higher tendency to form a micellar network that the linear counterparts.

Liu and coworkers [82] synthesized six-armed star polymers with identical arms made of poly(styrene) (PSt), poly (polyethylene glycol)actylate (PEG-A) and the block copolymer PSt-b-PEG-A utilizing the “core-first” approach via RAFT polymerization. At first, they prepared a six-arm multifunctional (hexa-functional) star RAFT agent that was then utilized for the synthesis of homo- and copolymer six-arm stars. The six-arm homostars were generated by using styrene or PEG-A, hexa-functional RAFT agent, AIBN as radical initiator and dioxane as solvent. The six-arm star block copolymers were synthesized by chain extension of the six-arm polystyrene with PEG-A. The obtained results from SEC and NMR measurements showed that the polymerization of six-arm star polymers is successfully mediated by the hexa-functional RAFT agent. Moreover, studies on the molecular weights of the synthesized six-arm star polymers demonstrated the compromised dynamic volume of six-arm star structure in respect to the linear precursors. In addition, the star polymers presented biodegradability due to the disulfide linkages between the arms and the core. It was also found that the star polymers degraded rapidly in the presence of DL-dithiothreitol (DDT) and degraded less rapidly in the presence of glutathione (GSH).

Liao et al. [83] prepared 4-armed stars made of 2-(dimethylamino) ethyl acrylate (DMAEA) monomer using the “core-first approach” via RAFT polymerization utilizing pentaerythritol tetrakis [2-(dodecylthiocarbonothioylthio)-2methylpropionate] (4-arm DDMAT) as the core-forming RAFT agent. In addition, they synthesized linear PDMAEA counterparts using 2- (dodecylthiocarbonothioylthio)-2-methylpropionic acid (DDMAT) as RAFT agent for comparison studies. They used similar RAFT agents for the synthesis of 4-arm star and linear polymers in order to maintain comparable synthetic routes and conditions as well as to control molecular weights of each structure. Star-shaped and linear polymers were successfully synthesized in two molecular masses (10Da and 20Da) as evidenced by SEC. Their aqueous solution properties, binding affinity to DNA, as well as their cytotoxicity towards cultured cell lines were also investigated. It was found that both star-shaped and linear PDMAEA polymers displayed aggregation tendency near the pKa of the PDMAEA, possibly because of the reduction in their solubility at higher pH values. Moreover, the star-shaped polymers at both molecular weights demonstrated better binding to DNA in comparison to linear counterparts and their cytotoxicity was not affected by the macromolecular architecture, but it was found to be solely influenced by the total molar mass.

Skandalis et al. [84] utilized the “core-first” approach to synthesize poly(2-(dimethylamino)ethyl methacrylate)-b-poly ((oligo ethylene glycol)methacrylate) (PDMAEMA-b-POEGMA)_n_ double-hydrophilic star block copolymers. Initially, they synthesized crosslinked core network using ethylene glycol dimethacrylate (EGDM) as the crosslinker, AIBN as radical initiator, 4-cyano-4-[(dodecylsulfanylthiocarbonyl)sulfanyl]pentanoic acid (CDTP) as CTA and 1,4 dioxane as the polymerization solvent. The polymerization of DMAEMA followed, utilizing the polymerized EGDM as the crosslinked macro-CTA core, AIBN as radical initiator and 1,4 dioxane as polymerization solvent, yielding PDMAEMA homostars. Subsequently, the synthesis of (PDMAEMA-b-POEGMA)_n_ double-hydrophilic star block copolymers was achieved using the PDMAEMA homostar as macro-CTA and OEGMA monomer. SEC curves displayed symmetrical peaks of (PDMAEMA-b-POEGMA)_n_ star blocks after purification that seems to result in final products of higher purity. Furthermore, DLS studies on the (PDMAEMA-b-POEGMA)_n_ double hydrophilic star blocks aqueous solutions showed that the star blocks aggregated at neutral pH, most probably due to the existence of hydrophobic EGDM cores.

Zhang et al. [85] applied a modified method based on two successive RAFT reactions in order to synthesize star polymers. Initially, they prepared branched copolymers with controlled degree of branching, variable chemical components and alterable CTA functionality via RAFT copolymerization of vinyl monomers such as methyl acrylate (MA), methylmethacrylate (MMA), styrene (St), or tert-butyl acrylate (tBA), using S-(4-S′-vinyl)benzylpropyltrithiocarbonate (VBPT) as the RAFT agent. The effects of monomer and initiator concentrations, comonomer feed ratio and reaction time on polymerization of VBPT RAFT agent with vinyl monomers were examined. It was found that the chemical composition of the end groups of branched copolymers, as well as the repeat units per branch could be adjusted by the reaction time and through control of feed ratio. They synthesized a series of branched copolymers with a wide range of molecular weights, variable CTA functionalities and branch numbers, which were used as multifunctional macro-RAFT agents for the synthesis of star-shaped polymers. Poly(VBPT-co-MA) branched copolymers as macro-RAFT agents were utilized for the polymerization of St or tBA monomers, and star-shaped polymers having branched cores with variable dispersities and controlled molecular weights were obtained. The synthesized star-shaped polymers and hyperbranched polymers were molecularly characterized by ^1^H-NMR and SEC-MALLS, indicating the success of the polymerization processes. Despite the fact that the arm number was not very precisely controlled, the synthetic approach based on two successive RAFT schemes is quite versatile and can be utilized for the synthesis of star-shaped polymers with alterable chemical components and number of arms similar to the original branched macro-CTAs.

## 3.2. “Arm-First” Approach

**Scheme 4:**
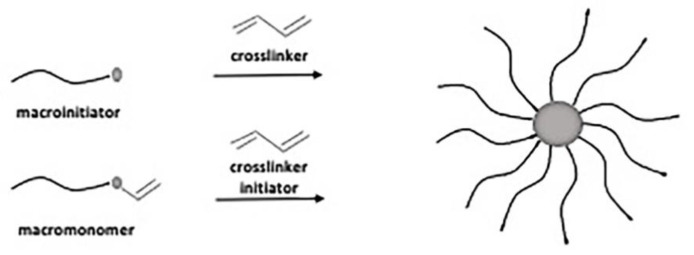
Schematic illustration of the “arm-first” approach.

The core-first approach often suffers from radical intermediate termination reactions and hence, optimization of the experimental conditions to eliminate these undesirable reactions become a significant consideration for producing star polymers of narrow dispersities (a parameter of great importance in biomedical applications) [7,10,31,35]. The initial step in the synthesis of star polymers via arm-first method, as the name suggests, is the preparation of polymeric arms (i.e., the macromolecular chain transfer agent or macro-CTA). Thereafter, the macro-CTA is chain extended with a di- or multi-functional vinyl monomer (crosslinker), resulting in the synthesis of star polymers. The arm-first strategy abrogates the need for generating multifunctional chain transfer agents and hence simplifies the synthetic procedures. However, the initial application of the RAFT polymerization for the synthesis of star polymers via arm-first strategy ordinarily led to star polymers with higher dispersities in comparison to those synthesized by the core-first approach, possibly owing to increased termination reactions deriving from the chain transfer process and the steric congestion [7]. The utilization of dispersed polymerization media (dispersions or emulsions) or a poorly soluble crosslinker can efficaciously ameliorate the dispersity to lower values, as first presented by Qiu et al. [86] and Ferreira et al. [87], respectively. These reaction schemes have become commonly used strategies for the preparation of star polymers utilizing the arm-first approach. However, there are some studies [88,89], controverting the necessity of dispersed systems or poor solvents, which potentially depend on the particular solvents, monomer and RAFT agent utilized. Furthermore, the molecular weight of the polymeric arms importantly affects the structure of the obtained star polymers. Higher molecular weight arms (longer in length) are confined to the formation of star polymers bearing a small core with a lower arm functionality, whereas lower molecular weight arms (shorter in length) favors the formation of star polymers carrying a large core with multiple short arms [7]. A schematic illustration of the “arm-first” approach is displayed in Scheme 4.

The latest achievements regarding the synthesis of star polymers by RAFT polymerization employing the “arm-first” approach are summarized below.

Boyer and his coworkers [90] utilized the arm-first method to produce biodegradable, cationic, well-defined poly-[dimethylaminoethyl methacrylate] (PDMAEMA) star polymers for the purpose of delivering siRNA to treat pancreatic and lung cancer cells. The synthetic strategy that the group followed was the preparation of PDMAEMA homopolymers via RAFT, which consisted the arms, and subsequently the chain growth in the presence of *N,N*-bis(acryloyl) cistamine and DMAEMA that both served as cross-linkers and star core formers. Finally, they obtained star polymers that fulfill the requirements of siRNA vectors. After the polymerization was completed, ^1^H-NMR spectroscopy revealed the full conversion of the crosslinker and the DMAEMA, while the arm consumption was determined to be around 80%. The number of the arms per star was determined by using the molecular weight estimated by light scattering. Each star was found to be comprised of ca. twenty arms. The complexation between the cationic amino groups of the PDMAEMA arms and the siRNA groups was achieved via electrostatic interactions. Ideal complexation between the two components was accomplished by increasing the star polymer ratio, while the siRNA was kept constant. The resulting complexes were well-defined, small in size and consistent in their physicochemical features. Cytotoxicity assays demonstrated that the complexes do not show any toxicity towards neither pancreatic nor lung cancer cells. Furthermore, the capability of the star polymers to deliver adeptly siRNA in both cell lines was confirmed and a substantial decrease in target gene mRNA and protein levels was observed. Finally, in vivo studies employing a mouse tumor model demonstrated that the delivery of clinically apt quantity of siRNA attached to the star polymer resulted in silencing the targeted gene expression by 50%.

Another important contribution towards the delivery of siRNA molecules for battling pancreatic tumors using star-branched vehicles was reported by Teo and her team [91]. They described the synthesis of poly-[2-(dimethylamino) ethyl methacrylate]/poly-[(oligo ethylene glycol)methacrylate] [PDMAEMA/POEGMA] mikto-arm stars via the “arm-first” methodology. Firstly, the PDMAEMA and POEGMA arms were synthesized via a typical RAFT polymerization process. PDMAEMA homopolymers of different lengths were prepared. For the formation of the core the crosslinker *N,N*-bis(acryloyl) cystamine and 2-(dimethyl amino)ethyl acrylate (DMAEA) was used. Finally, core crosslinked PDMAEMA/POEGMA stars were obtained. The PDMAEMA/POEGMA star shaped copolymers were soluble in water and showed enhanced stability in the aqueous medium. Self-assembly studies confirmed the successful complexation between the star polymers and the siRNA and the formation of uniform nanostructures. Furthermore, cell viability assays showed that the stars were nontoxic to healthy cells and accomplished to deliver siRNA with great efficacy to pancreatic cancer cells to silence TUBB3/ βIII-tubulin gene. The length of the PDMAEMA side-arms and the quantity of POEGMA impacted on both the internalization of stars into the pancreatic cancer cells and gene silencing efficiency. Moreover, the group proved that the embodiment of the POEGMA component is of high importance for permitting the star polymer to distribute and release siRNA into the cytosol and inhibit the gene expression unaffected by the serum proteins. Finally, in vivo studies showed that systemic administration of PDMAEMA/POEGMA star/ siRNA complexes led to high accretion of siRNA to pancreatic tumors in mice and inhibited the βIII-tubulin gene expression by 80%.

Skandalis et al. [84] reported on the synthesis of two more complicated mikto-arm stars composed of two different components via the “arm-first” strategy. The first one comprises the (poly- [dimethylaminoethyl methacrylate])_x_(poly-[lauryl methacrylate])_y_ PDMAEMA_X_PLMA_Y_ amphiphilic mikto-arm star, while the second one the PDMAEMA_n_POEGMA_n_ double-hydrophilic mikto-arm star. In both cases, ethylene glycol dimethacrylate (EGDM) difunctional monomer acted as the crosslinker and the core-forming component. For the formation of the PDMAEMA_x_PLMA_y_ amphiphilic mikto-arm stars PDMAEMA and PLMA homopolymers were prepared by RAFT and subsequently mixed and allowed to react with the cross-linker according to the aimed hydrophilic/ hydrophobic component ratio. Two different (PDMAEMA)x(PLMA)y amphiphilic mikto-arm stars were produced where one was of higher hydrophilic content while the second one of higher hydrophobic content. The apparent molecular weights (M_w app_) determined by static light scattering were larger than the ones determined by SEC due to the star-shaped structure of the copolymers. The number of the arms of each component was estimated from the molecular characterization data. For the preparation of PDMAEMA_n_POEGMA_n_ double-hydrophilic mikto-arm star, PDMAEMA homopolymer was synthesized first and then reacted with the EGDM resulting to the formation of a PDMAEMA homostar. In this way the CTA active groups were located inside the crosslinked core of the star. Subsequently, OEGMA addition led to the formation of PDMAEMA_n_POEGMA_n_ star-shaped copolymers. Similarly, with the case of PDMAEMA_x_PLMA_y_ mikto-arm stars, the apparent molecular weight was much higher than the molecular weight determined by SEC due to the architecture of the copolymer. Overall, the molecular characterization of the star copolymers confirmed star shaped macromolecular structures with adequate control in the molecular features of the copolymer materials through the synthetic paths followed. The group also performed post-polymerization reactions by using methyl iodide in order to quaternize the tertiary amine groups of the PDMAEMA components. Therefore, the star copolymers were converted into strong cationic polyelectrolytes of hydrophilic or amphiphilic character. Self-assembly investigations showed the formation of aggregates when the double-hydrophilic stars were dispersed in aqueous media and the formation of micellar structures when the amphiphilic stars were dispersed in water. The stars studied, excluding the quaternized ones, demonstrated a pH-responsive character in water due to the existence of the pH responsive PDMAEMA component. The complicated architecture along with the pH-responsive and positively charged nature of the star copolymers prompts their potential use in bioapplications.

Along the same lines, a different group has constructed a three-component star copolymer by implementing the “arm-first” approach. In particular, Wei et al. [92] combined three components of different chemical nature. Firstly, they synthesized separately, the cationizable poly[2-(dimethylamino)ethyl methacrylate] (PDMAEMA), the hydrophobic poly(n-butyl methacrylate) (PBMA) and the neutral hydrophilic poly[oligo(ethylene glycol) methacrylate] (POEGMA) homopolymers that eventually were utilized as the arms of the three-component star and the macro-RAFT agents. The preparation of the PDMAEMA/PBMA/POEGMA star was achieved by the combination of the three macro-RAFT agents along with the disulfide dimethacrylate (DSMA) redox-sensitive crosslinker bis(2-methacryloyl) oxyethyl disulfide in homogenous RAFT copolymerization. The authors discovered that the fragment of the crosslinker and hydrophilic/hydrophobic arm ratio affects greatly the control over the molar mass distribution. Specifically, the most promising results were obtained in the case where the PDMAEMA/POEGMA/PBMA ratio was 3:3:1 (M_w_/M_n_ < 1.3). Nevertheless, the molar mass distribution seemed to increase when the ratio DSDMA: total macro-RAFT agents or the fraction of the PBMA macro-RAFT increased. Subsequently, Wei’s team performed quaternization reactions with methyl iodide onto the PDMAEMA arms in order to produce strong cationic polyelectrolytes. In this manner, smart materials, able to be utilized as siRNA nanovectors were produced. The quaternized mikto-arm stars when inserted in aqueous media, formed uniform nanoparticles of an average size of 145 nm. Finally, the group reported on the successful fragmentation of the redox-responsive mikto-arm stars to the precursor linear arms by reduction with Bu_3_P.

Another important contribution, on the field of synthesis of star polymers via the “arm-first” strategy, aiming to potential utilization in cancer therapy, was made by Chen and his coworkers [93]. The mikto-arms stars presented in Chen’s work consisted of the same three arms PDMAEMA, PBMA and POEGMA as described above in the work of Wei et al. However, in this case the three different arms bear the RGD peptide-based RAFT agent. The preparation of the mikto-arm stars was conducted by the copolymerization of the three RGD-peptide arms along with the redox-sensitive disulphide dimethacrylate (DSDMA) crosslinker to finally get mikto-arms stars incorporating peptide groups. The optimal control over the molecular weight distribution and the highest yield were observed in the case of PDMAEMA: POEGMA:PBMA = 1:1:1 ratio. However, the yields of the star polymers containing peptide arms were relatively low. The group observed the formation of nanoparticles with sizes ca. 88 nm by DLS. The average size seemed to be unaffected by the increase of the arm molar ratio but an increase in the polydispersity index indicated low quality of the synthesized stars. The morphology of the nanoparticles was investigated by TEM, where larger nanostructures appeared (approximately 100 nm). The self-assembly into larger aggregates possibly is assigned to different study conditions, compared with DLS. In addition, cell viability investigations demonstrated that these type of star polymers exhibit low or negligible cytotoxicity. In summary, the multifunctional biorelevant star polymers proposed by Chen and his group hold significant properties such as enhanced biocompatibility, redox sensitivity, improved fluorescent properties and prospective aiming bioactivity, making them promising candidates for numerous bioapplications.

Equally interesting is the work of Li and his team [94], who reported the synthesis of polyzwitterionic star-branched 3-dimethyl(methacryloyloxyethyl) ammonium propane sulfonate (DMAPS) moderated by a macro-RAFT agent bearing carboxylic end-group, in the presence of *N*,*N*-methylenebis(acrylamide) (MBA) crosslinker, via the “arm-first” approach. The star-shaped polymer exhibits dual thermo- and pH-responsiveness. Unlike the linear PDMAPS arms, the UCST responsiveness of the star-shaped copolymers can be regulated by controlling the ratio of the macro-RAFT agent to the MBA crosslinker. The conversion of the arms into the star-branched copolymers was over 90%, while SEC measurements revealed the successful formation of star polymers with high molecular weight (M_n_ = 67.800 g/mol) and low molar mass distribution (1.13). Moreover, when the group investigated the UCST behavior by DLS, they observed that the hydrodynamic diameter of the formed nanoparticles was larger than the ones formed by the self-assembly of the linear arms, probably due to the branched configuration. In addition, the stars-shaped copolymers respond fast to pH changes along and present a notable and adaptable UCST range (above 36 °C from pH = 3 to pH = 10) due to the presence of numerous carboxylic acid end-groups. Rheological studies led the authors to the assumption that the star polymer was consisted of a rigid core and flexible arms. Consequently, the combination of pH and thermos-responsiveness of these polyzwitterionic star copolymers along with their rheological features makes them potential nanocarriers for drug delivery applications.

The preparation of another type of dual thermo- and pH-responsive stars was introduced by Sharker and his coworkers. [95] They reported on the synthesis of poly (*N*-isopropylacrylamide) (PNIPAM) star, as a result of the crosslinking of the PNIPAM linear arm bearing carboxyl end groups (-COOH) with the N,N’-methylenebisacrylamine (BIS). SLS measurements were conducted in order to determine the M_w_ of star and along with the M_w_ of the linear PNIPAM the number of the arms was defined and estimated to be 17. The pH and thermo-responsiveness of the star was investigated by DLS, SLS, UV-Vis spectrophotometry and TEM techniques. Light scattering studies showed that the thermo-induced character is in fact independent of pH for values below 5. Based on the data exported by the combination of the above-mentioned techniques the authors reached the conclusion that when the temperature is lower than the phase transition temperature (T_p_), at pH = 4, approximately two stars aggregate due to the existence of hydrophobic interactions. The star molecules form unimers at pH = 5. At pH = 10 the star chains show expansion. Above the T_p_, the stars formed compact aggregates with about 57 chains per aggregate. Under the same temperature conditions six polymers were aggregated at pH = 5, while polymer chains shrank at pH = 10. The morphology of the polymer aggregates was investigated by carrying out TEM measurements. Uncompleted spherical structures were noticed at pH 4, 5, and 10 (25 °C) and pH 10 (40 °C). In conclusion, the authors stated that the stability of the aggregated nanoparticles above the T_p_ renders them optimal tools for the formation of well-defined and regulated structures.

Ferreira et al. [87] proposed an optimal route towards the preparation of well-defined star polymers with low molar mass distribution. They used a variety of crosslinkers:(*N*,*N*-bis(acryloyl)cystamine,*N*,*N*-methylenebisacrylamide 1,2-dihydroxyethylene-bis-acrylamide and 1,6-hexanediol diacrylate in order to produce POEGA, PDMAEMA, Pt-BuA containing stars via the “arm-first” approach. Subsequently, they studied the effects of the solvent, the polymerization time, the crosslinker/macro-RAFT agent ratio, the nature of the crosslinker and the nature of the arm structure on the synthesis of star polymers. In order to examine the influence of the solvent, the reactant ratio was kept constant. DMF, toluene and acetonitrile were investigated using POEGMA arms. Well-defined stars of low molar mass distribution (M_w_/M_n_ < 1.2) with great arm embodiment ratio (higher than 90%) were obtained when *N*, *N*’-bis(acryloyl)cystamine was used as the core crosslinker in toluene. This was assigned to low solubility of the core in the poor solvent medium, resulting in nano-phase separation. For testing the influence of the polymerization time, the time of the crosslinking reactions ranged from 6 to 48 h, while the crosslinker/RAFT agent ratio was equal to 6 and 8. After 24 h of polymerization, shoulders on the SEC chromatograms appeared, indicating that after 24 h the polymerization should be stopped in order to obtain well-defined star polymers. As long as the crosslinker/ RAFT agent ratio was concerned, it was found that the arm incorporation increased as the crosslinker/ RAFT agent ratio increased. However, after the ratio reached the value of 16, macro-gels were formed. The narrowest polydispersity index was obtained when the crosslinker/ RAFT agent ratio was equal to 8 and the *N*, *N*’-bis(acryloyl)cystamine was the crosslinker. Moreover, when the *N*, *N*’-methylene bisacrylamide was used as the crosslinker and the [crosslinker]: [RAFT AGENT] ratio was equal to 4, the best incorporation of arms into star-shaped structures along with the lowest Ð were obtained. After the synthetic approach of POEGMA star based polymers using soluble crosslinkers was optimized, PNIPAM, POEGA and Pt-BuA stars were synthesized using *N*, *N*’-bis(acryloyl)cystamine as the crosslinker. Ratios of [crosslinker]_0_:[RAFT agent]_0_ equal to 4: 1 and 8: 1 led to stars with higher than 90% incorporation of the arms and to dispersity indexes lower than 1.2. In conclusion, the authors declared that very well-defined star polymers with low polydispersity, can be produced utilizing the ‘arm-first’ strategy and RAFT polymerization, incorporating higher number of arms, when poorly soluble crosslinked cores are formed. In contrast, crosslinked cores completely compatible with the solvent utilized, result in significantly decreased incorporation of arms into the star-branched structures which show broad molecular weight distributions.

Bray et al. [96] synthesized multiblock core crosslinked star polymers of both homopolymers and copolymers of 2-acrylamido-2-methylpropane sulfonic acid (AMPS) with either *N*-hydroxyethylacrylamide (HEAm) or 4-acryloylmorpholine (NAM) as a comonomer, using the arm-first approach via RAFT polymerization. At first, they prepared a linear polymer which was then utilized as the first arm, followed by the addition of a crosslinker in a one-pot step. At the beginning, the effect of crosslinker to CTA ratio was examined by employing *N*, *N*′-methylenebisacrylamide (C1) as the crosslinker, and homopolymer PAMPS_50_ as the model arm. They found that the crosslinker to CTA ratio of 8 was the optimal as the synthesized star polymer based on the specific ratio presented low dispersity (1.24), high arm incorporation (90%), high molecular weight (64 kg/mol) and no visible radical-radical coupling. Having determined the optimal crosslinker to CTA ratio, they then studied the effect of the crosslinker structures using a series of crosslinkers with different solubility in aqueous media and functionalities, such as divinylbenzene (C2, aromatic crosslinker), N, N′-Methylenebis(acrylamide) (C3, hydrophilic acrylamide crosslinker) and ethylene glycol diacrylate (C4, acrylate crosslinker). The results from SEC measurements showed that the acrylate crosslinker (C4) gave the narrowest chromatographs that could be attributed to the lower reactivity of acrylates in comparison to acrylamides. Crosslinker C2 resulted in lower molecular weight stars compared to the stars synthesized utilizing either crosslinkers C1 or C4 and this fact could be explained by the poor solubility of C2 in aqueous media that confined the incorporation of the arms to the star. In the case of crosslinker C3, no star polymer formation was observed, as evidenced by SEC. In addition, they investigated the effect of arm length on the synthesis of star polymers. They used crosslinker C4, keeping constant the crosslinker to CTA ratio at 8, while altering the molecular weight of the AMPS homopolymer arm from 11 kg/mol to 35 kg/mol. The efficiency of the polymerization reaction decreased as the arm length increased and the incorporation of the arm into the star was reduced, due to either the increase of steric hindrance or the increased viscosity of the reaction solution. Moreover, they studied the effect of arm composition by synthesizing star copolymers made of AMPS and HEAm monomers, namely octablock, diblock and random copolymers. In particular, the synthesis of star polymers utilizing an arm consisted of diblock copolymer resulted in a gel-like polymer that could not be analyzed by SEC. The authors attributed this fact to the high concentration of crosslinker utilized. When employing random copolymer, a star polymer with high arm incorporation and molecular weight of 100 kg/mol was obtained, whereas when using the octablock, a star polymer with a high molecular weight (180 kg/mol) but low arm incorporation was obtained. In conclusion, Bray et al. synthesized multiblock crosslinked stars utilizing the “arm-first” approach via RAFT polymerization and deduced that the crosslinker to CTA ratio, as well as the structure of the crosslinker are important parameters for the synthesis of well-defined star polymers with high arm incorporation.

Vlassi et al. [97] reported on the synthesis of amphiphilic mikto-arm copolymers with mixed arms of poly (lauryl methacrylate) (PLMA) and poly (oligo ethylene glycol methacrylate) (POEGMA) employing arm-first approach via RAFT polymerization technique. At first, they synthesized the homopolymers of PLMA and POEGMA using 4-cyano-4-(phenyl-carbonothioylthio) pentanoic acid (CPAD) as chain transfer agent, AIBN as radical initiator and dioxane as polymerization solvent. Subsequently, two mikto-arm PLMA_x_POEGMA_y_ star copolymers having different mass content of the components were prepared utilizing PLMA (hydrophobic arm) and POEGMA (hydrophilic arm) homopolymers, ethylene glycol dimethacrylate (EGDM) as the crosslinker and dioxane as the solvent. SEC curves displayed monomodal and symmetrical peaks of PLMA_x_POEGMA_y_ mikto-arm star copolymers and the results extracted from ^1^H-NMR and FT-IR measurements confirmed their chemical structures. Moreover, DLS studies on the mikto-arm star copolymers aqueous solutions showed that the star copolymers formed supramolecular aggregates with nanoscopic dimensions. In addition, the formed aggregates can successfully encapsulate curcumin (a hydrophobic drug molecules with intrinsic fluorescence) and the reorganization of the encapsulated nanostructures depended on the curcumin content and the composition of star copolymers. Conclusively, the synthesized amphiphilic mikto-arm star copolymers formed nanoaggregates in aqueous media with potential use in drug delivery and bioimaging applications.

## 4. Conclusions

The achievements discussed in the previous sections clearly demonstrate that the RAFT polymerization process can be successfully utilized for the synthesis of ABC linear triblock terpolymers and star polymers, with controlled macromolecular structures, which can incorporate a large and diverse number of chemical functionalities. This diversity in composition allows for obtaining polymers with very interesting self-assembly properties able to act as nanocarriers and nanovehicles, important for biorelated applications, with special focus on drug delivery and bioimaging. Although the field has already shown significant progress further advances are expected in the future which will allow for the synthesis of more complex macromolecular structures with embedded physicochemical and self-organizing properties, which will facilitate the development of smart and functional polymeric materials.

## Data Availability

Not applicable. No data have been created.
